# Supplementary material for: A Cancer-Specific Anti-Podoplanin Monoclonal Antibody, PMab-117-mG2a Exerts Antitumor Activities in Human Tumor Xenograft Models
Source: Cells. 2024 Nov 6;13(22):1833. doi: 10.3390/cells13221833 (PMC11593084; doi:10.3390/cells13221833)
Supplement: Supplementary file 1 [file cells-13-01833-s001.zip › cells-3270758-supplementary.pdf]

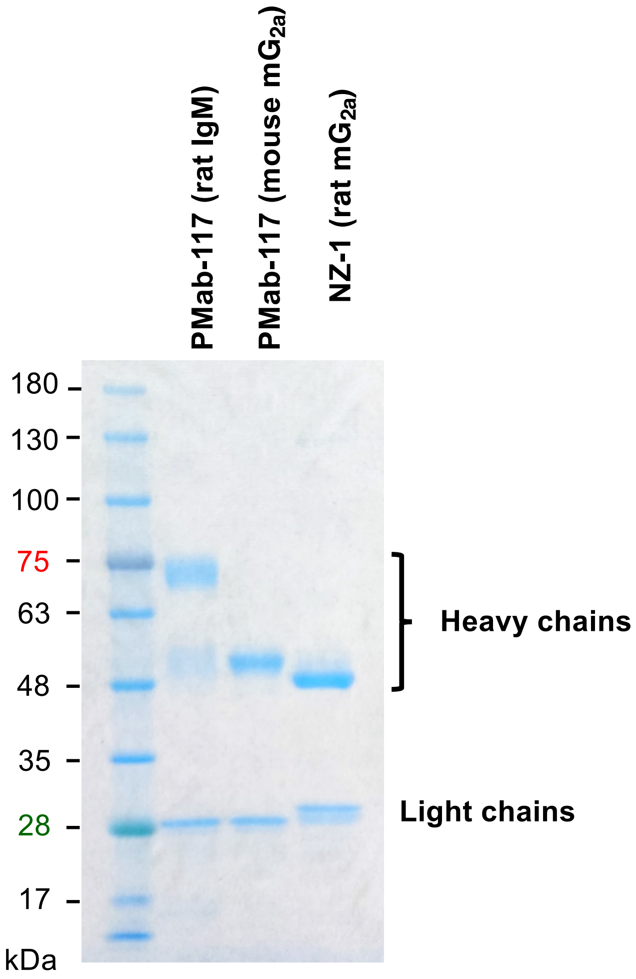

Supplementary Figure S1. Confirmation of the purified mAbs.
MAbs (2 μg) were treated with sodium dodecyl sulfate (SDS) sample buffer containing 2-mercaptoethanol (Nacalai Tesque, Inc.). Proteins were separated on 5%–20% polyacrylamide gel (FUJIFILM Wako). The gel was stained by Bio-Safe CBB G-250 Stain (Bio-Rad Laboratories, Inc.).

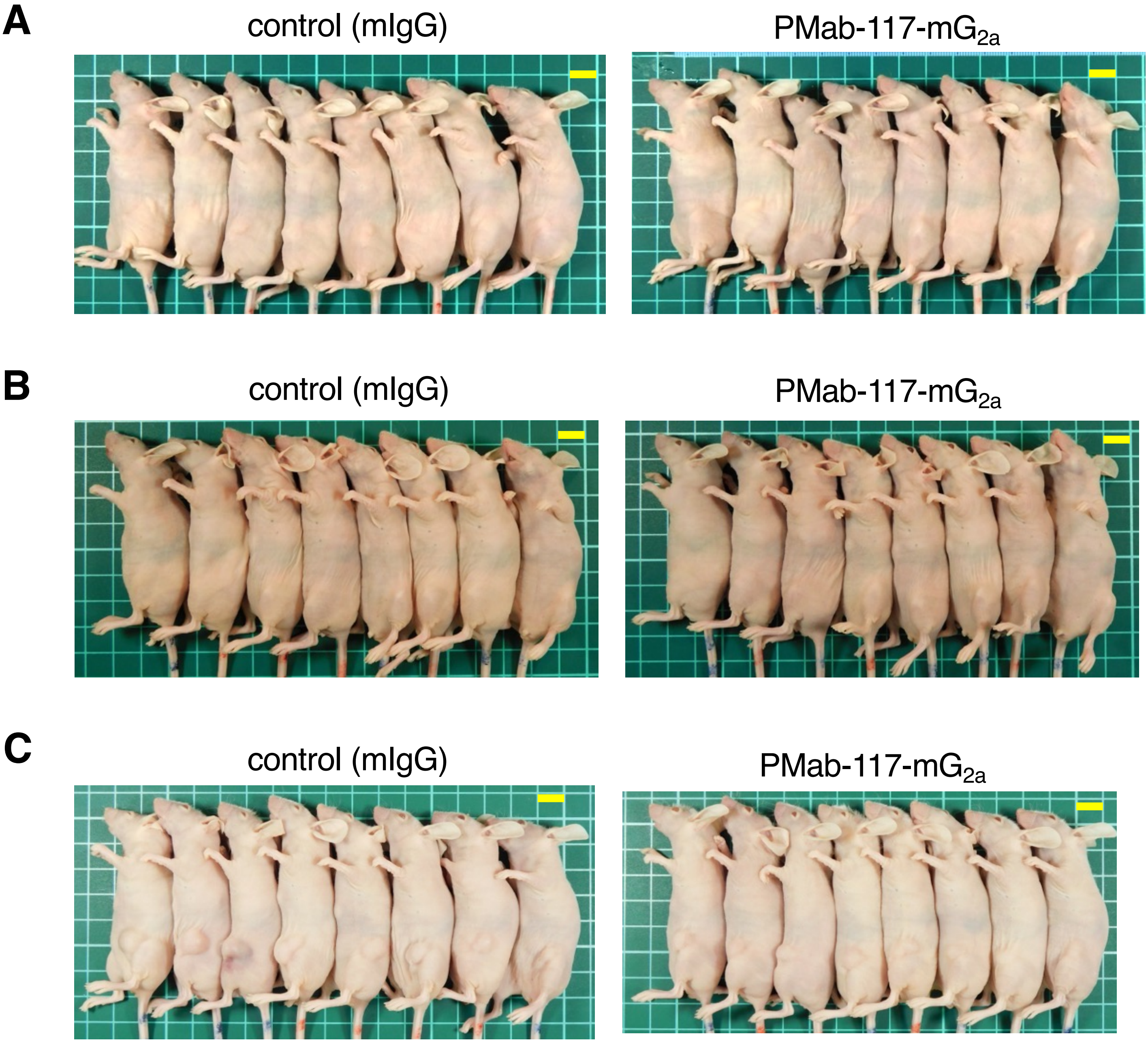

Supplementary Figure S2. Body appearance in LN229/PDPN (A), PC-10 (B), and LN319 (C) xenografts-implanted mice treated with control mIgG or PMab-117-mG$_{2a}$ on day 30 (LN229/PDPN) , day 28 (PC-10) or day 22 (LN319) after cell inoculation. Scale bar, 1 cm.
